# Macrodystrophia lipomatosa involving multiple nerves

**DOI:** 10.1007/s10195-011-0159-6

**Published:** 2011-09-27

**Authors:** Seema Rohilla, Nitin Jain, Rambaksh Sharma, Dhara B. Dhaulakhandi

**Affiliations:** 1 Department of Radiodiagnosis and Imaging, Postgraduate Institute of Medical Sciences, Pt. B.D. Sharma University of Health Sciences, Rohtak, 124001 Haryana India; 2 Department of Biotechnology and Molecular Medicine, Postgraduate Institute of Medical Sciences, Pt. B.D. Sharma University of Health Sciences, 1st Floor, Regional Cancer Center, Rohtak, 124001 Haryana India

**Keywords:** MDL, Multiple nerve territories, Proteus syndrome

## Abstract

Macrodystrophia lipomatosa (MDL), a rare congenital disorder, is considered by some to be a localized form of Proteus syndrome. The implication of the PTEN (phosphatase and tensin homolog deleted on chromosome 10) gene in both strengthens this belief. We present a case who had MDL in multiple nerve territories—all on the same side of the body—with hypertrophy of mainly fibroadipose tissue throughout their distribution, thus pointing to a form of localized hemihypertrophy; both hemihypertrophy and lipomatous tumors are components of Proteus syndrome.

## Introduction

Macrodystrophia lipomatosa is a congenital, progressive, nerve territory oriented, localized gigantism. There is proliferation of all mesenchymal elements, but especially adipose tissue. The abnormal growth ceases at puberty, but the overgrown part is prone to joint degeneration and compression of the neurovascular bundle in some patients. It needs to be differentiated from other conditions such as hemangioma, lymphangioma, or neurofibromatoses. Rarely, it involves multiple nerve territories. We present one such case of gigantism in multiple nerve territories.

## Case report

**Fig. 1:**
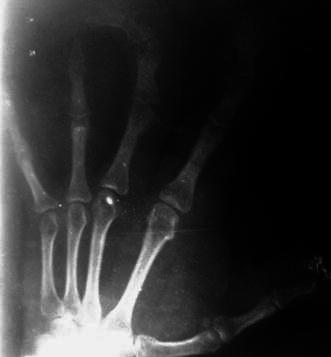
Plain radiograph (AP view) of the left hand showing enlargement of the phalanges of the thumb and the index and middle fingers, along with soft tissue hypertrophy

**Fig. 2:**
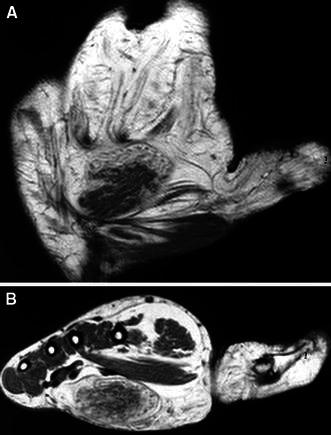
T1 W coronal (**a**) and axial (**b**) images of the left hand, showing soft tissue hypertrophy and an enlarged median nerve, with fat infiltrating the nerve

**Fig. 3:**
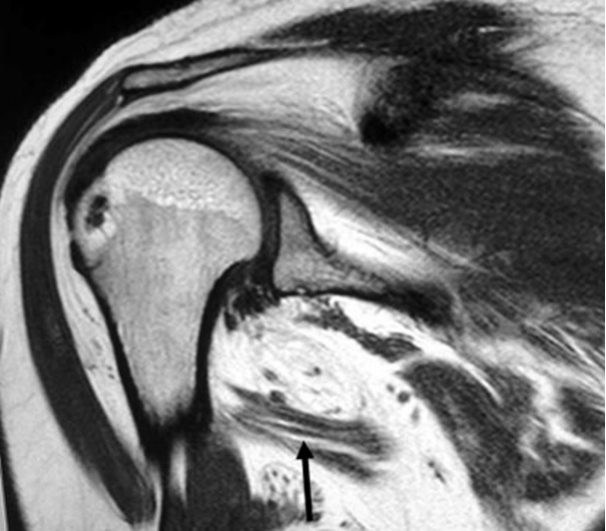
T1 W coronal oblique image of the left shoulder, showing enlargement of the axillary nerve in the quadrilateral space (*arrow*) with fatty infiltration of the nerve

**Fig. 4:**
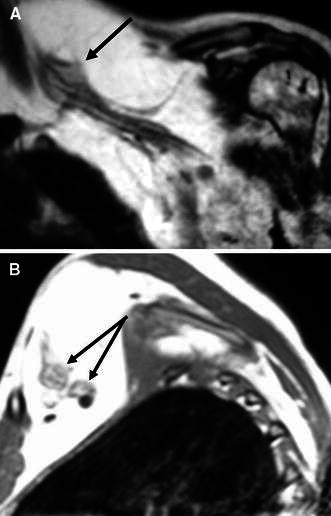
T2 W coronal (**a**) and T1 W sagittal (**b**) images of the left brachial plexus, showing enlargement of all three trunks (*arrows*) along with fatty infiltration

**Fig. 5:**
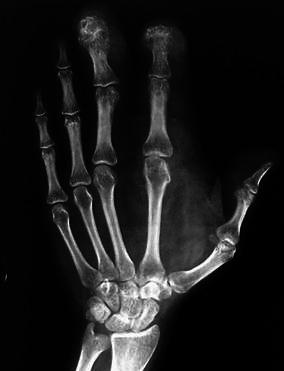
Postoperative plain radiograph (AP view) of the left hand showing partial amputation of the overgrown digits (middle phalanges of the second and third digits have been removed) and reduced soft tissue bulk

A 20 year old female presented with painless enlargement of the lateral aspect of the left hand, left thumb, the index and middle fingers, and also of the left shoulder, which had been present since childhood. The skin over these digits was thick and non-tender. There were no associated nodules, cafe-au-lait spots, or pitting edema, and no audible bruits or thrills. Plain radiograph showed enlargement of the phalanges of the thumb, index, and middle fingers, along with soft tissue hypertrophy (Fig. 1) and soft tissue enlargement about the left shoulder. MRI examination revealed hypertrophy of soft tissue (mainly fat) of the first, second and third digits, mainly on the volar aspect, along with fusiform enlargement of the median nerve just distal to the carpel tunnel (Fig. 2a, b). There was fatty infiltration of the nerve with separation of nerve fibres. No enhancement was seen on contrast enhanced MR. Similar fatty infiltration was seen along the second and third digital nerves. There was hypertrophy of soft tissue (mainly fat) in the region of the shoulder, along with fatty infiltration of the supraspinatus, infraspinatus, and teres minor muscles, and the posterior half of the deltoid muscle. The axillary nerve was enlarged and showed fatty infiltration in the quadrilateral space (Fig. 3), the left brachial plexus showed similar masses involving all (upper, middle and lower) trunks, along with hypertrophy of soft tissue (mainly fat) in this area (Fig. 4a, b). Our patient appeared to have a combination of fibrolipomatous hamartoma (FLH) and macrodystrophia lipomatosa, though it is very difficult to separate these two entities, as detailed in the underlying discussion. The patient was asymptomatic but underwent surgery for cosmetic reasons. The soft tissue bulk was reduced and partial amputation of the overgrown digits was done. The middle phalanges of the second and third digits were removed, while the distal phalanges were retained (to preserve nails) (Fig. 5). The patient provided her consent to the publication of this case report.

## Discussion

First described by Feriz in 1925, macrodystrophia lipomatosa is an unusual form of local hypertrophy, congenital in origin but not hereditary [1–3], and characterized by proliferation of all mesenchymal elements but with a disproportionate increase in adipose tissue. It is a form of true macrodactyly where the degree of overgrowth of the affected digits/limb is faster than the normal growth pattern, and such abnormal growth ceases at puberty. The abnormality corresponds to the zone of innervation by sclerotome. It is mostly unilateral; bilateral involvement is rare [4]. Involvement of a lower limb is more frequent than an upper limb, and the most common sites are the second and third digits [5].

FLH of the nerve is a rare tumor-like condition that consists of an infiltration of connective tissue and fatty elements of the nerve. FLH produces digital overgrowth and can be confused with MDL. FLH of the nerve may occur without macrodactyly, but those with macrodactyly are the same as MDL [6]. It usually presents as an isolated nerve lesion and associated intramuscular fat deposition [7], while in MDL, fat deposition also occurs in periosteum, leading to bony changes in addition to fat deposition in nerve sheath, subcutaneous, and muscle compartments [8]. The enlargement of the axillary nerve and all of the cords of the brachial plexus in our patient likely represents typical FLH according to the above description, while enlargement of the hand and three digits represents MDL, because there is no bony overgrowth of the humerus, while there is bony overgrowth of the second and third metatarsals and phalanges. Moreover, a combination of FLH and MDL of the median nerve is reported to exist [9], as in our case. Skip lesions at the median nerve of the middle forearm have been described [9]. The FLHs involving three cords of the brachial plexus, axillary nerve, median nerve, and digital nerves in our case may also represent such skip lesions.

Macrodystrophia lipomatosa is not a hereditary disorder. Its etiology is uncertain. Various hypotheses include lipomatous degeneration [1], disturbed fetal circulation [2], erroneous segmentation [2], the trophic influence of a tumified nerve [1], and the in utero disturbance of a growth-limiting factor [3]. The condition is largely asymptomatic, but localized overgrowth can lead to deformities that can hinder function. Moreover, due to deformities secondary to the localized overgrowth, there are premature degenerative changes in joints. There may be compression of a neurovascular bundle. The patient seeks consultation for either cosmetic reasons or due to functional impairment due to deformity or degenerative changes. Rarely, symptoms may be due to neurovascular compression, with surgery required.

The corrective surgery aims to reduce the bulk of the overgrown fibrofatty tissue and to partially amputate the overgrown digits. There is a localized recurrence rate of 33–60% in macrodystrophia lipomatosa [6]. Our patient underwent surgery of the hand, which reduced the bulk of the soft tissue, and the overgrown digits were partially amputated (middle phalanges of the second and third digits were removed).

Histology of the lesion shows a massive increase in adipose tissue interspersed with a fine mesh of fibrous tissue that involves subcutaneous tissue, bone marrow, periosteum, muscle, and nerve sheaths. The integrity of the trabecular architecture remains normal. The soft tissue growth is more marked on the volar aspect, leading to dorsal deviation of the involved digits, as in our case. Macrodystrophia lipomatosa should be distinguished from other congenital anamolies such as hemangioma, lymphangioma, Ollier’s disease, Klippel–Trenaunay–Weber syndrome, and plexiform neurofibromatosis (NF). Among these, hemangioma, lymphangioma, and Klippel–Trenaunay–Weber syndrome can be easily distinguished based on ultrasound and signal characteristics on MRI. Hemangiomas show a septate configuration of high signal intensity channels on T2 W images corresponding to the vascular channels and fibrous strands. Lymphangiomas are hyperintense to muscle on T1 W and hyperintense to fat on T2 W images. MR images show malformed venous and lymphatic lesions as areas of high signal intensity on T2 W images in Klippel–Trenaunay–Weber syndrome, and depict the deep extension of low-flow vascular malformations into muscular compartments and the pelvis. Ollier’s disease can also be easily made out on X-rays of the affected part. Plexiform neurofibromatosis is most difficult to distinguish from macrodystrophia lipomatosa, but unlike NF, macrodystrophia lipomatosa does not show familial occurance or cutaneous stigmata. In addition, there are thinned bone shafts and waxy cortices in NF, while in MDL there is bony enlargement [10].

As mentioned previously, MDL is a nonhereditary congenital localized gigantism with skeletal abnormalities and a marked disappropriate increase in fibroadipose tissue. Some authors suggest that MDL is a localized form of Proteus syndrome [11, 12]. Though the exact etiology is unknown, the disease is believed to be due to the alteration of somatic cells during limb bud development and disturbed fetal circulation [13]. Recent genetic studies have indicated involvement of the PTEN (phosphatase and tensin homolog deleted on chromosome 10) gene, which is primarily a tumor suppressor gene and substantially influences cell/tissue growth [14]. PTEN mutations and PTEN nullizygosity have already been reported in Proteus and Proteus-like syndromes, which—in addition to hemihypertrophy, lower limb asymmetry, and arteriovenous malformations—also presents with lipomatosis [15, 16]. Although the regulatory function of the PTEN gene in cell growth is well established, which possibly accounts for the marked increase in adipose tissue mass, more evidence on the molecular genetic side is needed to categorically prove the role of the PTEN gene in MDL, and more specifically the mechanism of disproportionate lipomatous formation [17, 18, 19].

Macrodystrophia lipomatosa may be considered a part of Proteus syndrome, a generalized hamartomatous disorder involving hemihypertrophy consisting of lipomatous tumor in addition to other features, such as skull anamolies, pigmented naevi, lung cyst, and intra-abdominal lipomas [20–22]. Our case corroborates this fact, as all the hypertrophied parts and nerve hamartomas are on the same side of the body (i.e., the left side), thus pointing to localized hemihypertrophy along with lipomatous tumors.

MDL and FLH are two entities that are closely related and difficult to dissociate, as in our case. It is important to diagnose macrodystrophia lipomatosa as the condition is initially progressive but stabilizes at puberty. Correct diagnosis obviates unnecessary anxiety, and corrective surgery at the appropriate stage may help the patient. The involvement of the PTEN gene in the etiology of both MDL and Proteus syndrome suggests that MDL is a localized form of Proteus syndrome. The unilateral involvement of all nerve territories in our patient provides physical evidence of this fact, as it suggests localized hemihypertrophy, a component of Proteus syndrome.
